# Intermittent White Urine With Nephrotic-Range Proteinuria and Preserved Renal Function: A Diagnostic Dilemma Between Chyluria, Pseudochyluria, and Amyloidosis

**DOI:** 10.7759/cureus.102810

**Published:** 2026-02-02

**Authors:** Firdaus Jabeen, Vaibhav Shukla, Salil Vallecha, Monis Khan, Saboor Mateen

**Affiliations:** 1 Internal Medicine, Era's Lucknow Medical College and Hospital, Lucknow, IND

**Keywords:** amyloidosis, chyluria, nephrotic-range proteinuria, pseudochyluria, white urine

## Abstract

Milky or “white” urine is usually attributed to chyluria but may also result from lipid-rich proteinuric urine or crystals (“pseudochyluria”). We describe a 47-year-old woman with a 6-month history of intermittent white urine, bilateral edema, nephrotic-range proteinuria (20.3 g/24 h), preserved renal function, near-normal serum albumin, and chronic bronchiectasis. Evaluation for parasitic and non-parasitic chyluria, tuberculosis, and systemic amyloidosis was repeatedly negative. A fasting urine sample showed markedly raised triglycerides but negative tests for chyle. The kidney biopsy was non-representative, and the abdominal fat-pad biopsy was Congo-red-negative. Serum anti-PLA2R antibodies and free light chains were normal. The case illustrates the gray zone between chyluria and pseudochyluria and the challenge of defining the underlying glomerular lesion when tissue diagnosis is limited.

## Introduction

Chylous urine most often reflects a lymphatico-urinary fistula from filarial disease or non-parasitic lymphatic obstruction and can mimic nephrotic syndrome when accompanied by edema and heavy proteinuria [1,2]. Turbid white urine may also arise from lipid-rich nephrotic urine, infection, or crystals (“pseudochyluria”) [2,3]. Chronic bronchiectasis is a recognised cause of secondary (AA) renal amyloidosis presenting with proteinuria and progressive renal dysfunction [4]. In settings where filariasis, tuberculosis, and chronic infections are endemic and access to repeat renal biopsy or mass spectrometry is limited, distinguishing chyluria, pseudochyluria, and amyloidosis can be difficult. We report a patient with intermittent white urine, massive proteinuria, preserved renal function, and bronchiectasis in whom extensive evaluation for chyluria and amyloidosis remained negative.

## Case presentation

A 47-year-old woman presented with 6 months of intermittent, strikingly white, milky urine and progressive bilateral pitting pedal edema. There was no dysuria, colicky pain, fever, weight loss, or known lymphatic disease. She had a several-year history of chronic cough with intermittent purulent sputum; earlier cultures had grown mixed bacteria and *Candida* species. Screening for secondary immunodeficiency (HIV, glycemic status, and complete blood counts) was normal, and there was no history of recurrent severe infections or immunosuppressive therapy.

**Figure 1:**
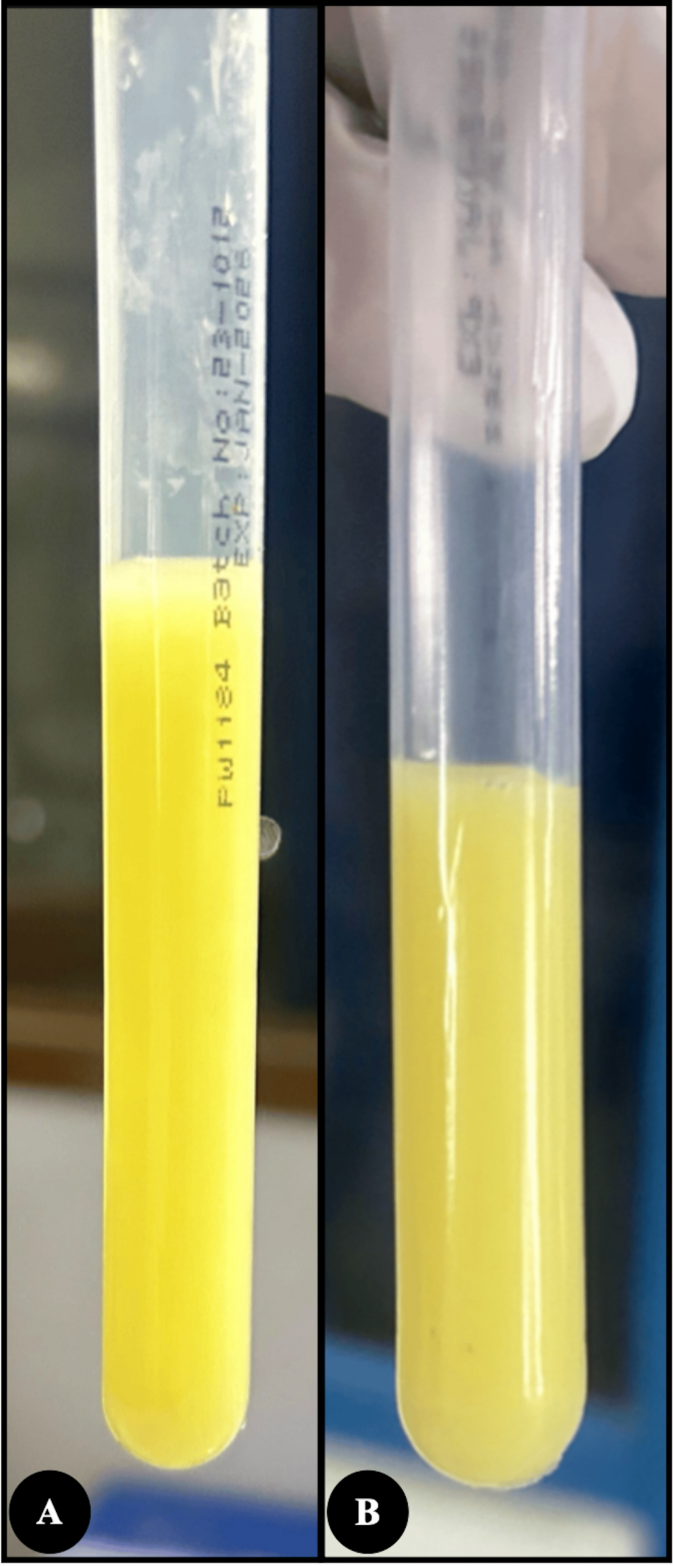
Physical appearance of urine (A) Initial sample showing dense, white-yellow, turbid urine at presentation; (B) Follow-up sample after six months showing persistent white-yellow turbidity

Blood pressure and systemic examination were unremarkable, apart from edema. Initial investigations showed a normal complete blood count and liver and renal function tests. A 24-h urine sample demonstrated nephrotic-range proteinuria of 20.3 g/day. Spot urinalysis repeatedly revealed turbid urine, 2+-3+ protein, microscopic hematuria, and pyuria without casts (Table 1, Figure 1).

**Table 1:** Serial hematological, biochemical, and urinary parameters from presentation (Day 1 to Day 240), showing persistent nephrotic-range proteinuria and intermittent turbid white urine with preserved renal function and stable systemic inflammatory markers WBC: White blood cells; INR: International normalized ratio; LDH: Lactate dehydrogenase; HDL: High-density lipoprotein; VLDL: Very low-density lipoprotein; LDL: Low-density lipoprotein; RBC: Red blood cells; AFB: Acid-fast bacilli; C/S: Culture and sensitivity; GPC: Gram-positive Cocci; GNB: Gram-negative bacteria. “++” and “+++” denote semi-quantitative dipstick grading of urine protein (moderate and heavy proteinuria, respectively), and “–” indicates that the test was not performed.

Test	Day 1	Day 30	Day 60	Day 90	Day 120	Day 180	Day 240	Normal Range
Hemoglobin (Hb)	15.4	13.5	12.5	12.6	12.2	13.0	12.3	12.0 to 15.5 g/dL
Total Leucocyte Count (WBC)	10600	10000	7000	7500	8500	11000	7800	4000-11000 Cells/cumm
Platelet Count	4.2	3.0	3.0	2.8	2.8	3.2	2.8	1.5 - 4.5 Lakh /cumm
Blood Urea	20	22	21	14	20	22	11	6 to 21 mg/dL
Creatinine	0.7	0.5	0.7	0.7	0.6	0.6	0.6	0.6 to 1.1 mg/dL
Serum Bilirubin (Total)	1.1	1.0	0.6	0.9	0.5	1.0	1.1	0.2 - 1.3 mg/dl
Prothrombin Time	12.5	12.5	12.2	11.7	11.9	12.0	11.3	9.8-12.1
INR	1.1	1.1	1.07	1.0	0.91	1.02	1.02	0.6-1.5
Serum Calcium	8.5	8.6	8.4	8.2	7.8	8.4	8.9	8.6-10.2 mg/dl
Serum Phosphorus	3.5	3.6	3.4	3.2	3.6	3.7	3.8	2.5-4.5 mg/dl
Total Protein	6.1	5.9	6.4	7.0	8.2	7.0	6.1	6.4-8.3 g/dl
Serum LDH	245	-	-	-	-	-	236	120-246 U/L
Serum Albumin	3.4	3.2	3.6	3.7	4.7	3.8	3.6	3.5-5.2 g/dl
Serum Triglycerides	138	184	-	-	-	-	144	< 161.0 mg/dl
Serum Cholesterol	173	206	-	-	-	-	201	200-239 mg/dl
Serum HDL Cholesterol	65	70	-	-	-	-	54	42-88 mg/dl
Serum VLDL Cholesterol	28	37	-	-	-	-	20	2-30 mg/dl
Direct LDL Cholesterol	98	120	-	-	-	-	101	<100 mg/dl
C- Reactive Protein	<5	-	-	-	7.7	8.8	<5	<10 mg/dl
Urine Appearance	Turbid White	Turbid White	Dark Yellow	Pale Yellow	Turbid White	-	Turbid white	None
Urine Protein	+++	+++	+++	++	+++	-	+++	None
Urine Epithelial Cells	Occasional	Occasional	2-4/hpf	2-4/hpf	Occasional	-	Occasional	None
Urine Pus Cells	24-24/hpf	Occasional	2-4/hpf	8-10/hpf	Occasional	-	Occasional	None
Urine RBC	Plenty/hpf	Nil	Nil	Nil	Plenty/hpf	-	Nil	None
Urine Sugars	Nil	Nil	Nil	Nil	Nil	-	Nil	None
Urine Cast/Crystals	Nil	Nil	Nil	Nil	Nil	-	Nil	None
Urine for Dysmorphic RBC	-	-	-	Not Detected	-	-	Not Detected	Not Detected
Urine Microalbumin	-	6.77		705	-	-	-	0-20 mg/L
Urine Protein		-		142	-	-	-	0-14 mg/dL
Urine Creatinine	-	74.30		92	-	-	-	28-217 mg/dl
Urine Albumin-Creatinine Ratio	-	9.11		766	-	-	-	0-0.20 mg/g creatinine
Urine Protein/Creatinine Ratio	-	-		1.33	-	-	-	0.00-0.20
Urine Triglycerides				55		-	-	< 10 mg/dl
Urine Chyle	Absent	Absent	Absent	Absent	Absent	Absent	Absent	Absent
24-Hour Urinary Protein	21000	20304	-	-	-	-	18000	42 - 225 mg/day
Urine Culture	Sterile	Sterile	Sterile	Sterile	Sterile	Sterile	Sterile	Sterile
Sputum AFB (2 Days)	Negative	-	Negative	-	-	Negative	-	Negative
Sputum C/S	Growth of a non-pathogenic organism	-	Growth of a non-pathogenic organism	-	-	-	-	Growth of a non-pathogenic organism
Sputum Gram Stain	Pus cells >25, epithelial cells 10-25, numerous GPCs in clusters, chains, pairs, moderate GNB seen	-	No pus cells or epithelial cells, numerous GPCs in clusters, chains, pairs	-	-	-	-	Sterile
Urine AFB	Negative	-	Negative	-	-	-	-	Negative

**Figure 2:**
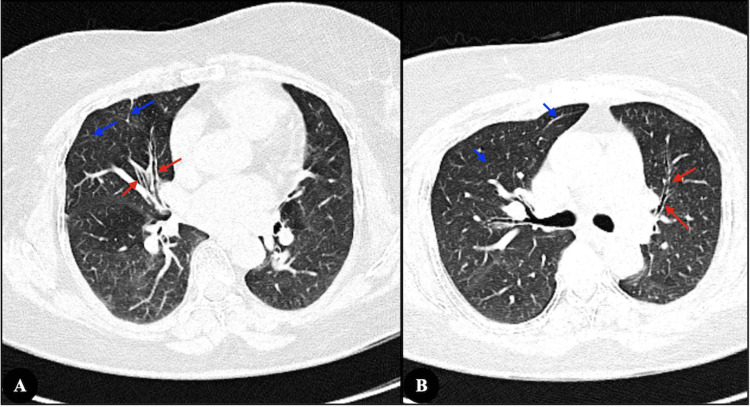
High-resolution computed tomography of the chest (A) Traction bronchiectasis in the medial segment of the right middle lobe (red arrows) with adjacent fibrotic streaks (blue arrows); (B) Traction bronchiectasis in the superior lingular segment of the left upper lobe (red arrows) with associated fibrotic bands (blue arrows)

Ultrasonography showed normal-sized kidneys with preserved corticomedullary differentiation and mobile linear echogenic strands in the bladder, raising the possibility of chyluria. CT abdomen was otherwise normal. High-resolution CT chest demonstrated bilateral lower-lobe air trapping, fibrotic bands with traction bronchiectasis in the right middle lobe and lingula, and calcified mediastinal nodes, consistent with long-standing post-infective bronchiectasis (Figure 2).

Urine for chyle was negative on repeated testing, including an early-morning fasting sample. In the same fasting specimen, urinary triglycerides were markedly elevated (55.9 mg/dL; reference <10 mg/dL). Filarial antigen, urine, and sputum AFB smears, urine filarial antigen testing, urine and sputum AFB smears, urine GeneXpert MTB/RIF assay (cartridge-based nucleic acid amplification test), and Mantoux test were all negative; an interferon-γ release assay (QuantiFERON-TB Gold) could not be performed because of financial constraints. A native kidney biopsy was non-representative, with only scant renal cortex (three preserved glomeruli) and no immune deposits on immunofluorescence (IgG, IgM, IgA, C3, C1q, κ, λ all negative). Serum free light chains were within the normal ratio (κ 20.9 mg/L, λ 14.9 mg/L, κ/λ 1.40), and serum anti-PLA2R antibodies were negative (<2 RU/mL). Abdominal fat-pad biopsy was negative for amyloid on Congo red staining, and serum amyloid A was just above the normal range. After counselling about further options, the patient declined a repeat renal biopsy or additional invasive procedures.

She was treated conservatively with telmisartan 40 mg once daily, dapagliflozin 5 mg once daily, torsemide 10 mg on alternate days, and dietary sodium restriction. Pedal edema improved within a few weeks and remained controllable with intermittent low-dose diuretics. During approximately nine months of outpatient follow-up, serum creatinine stayed stable at 0.6-0.8 mg/dL and serum albumin rose from 3.2 g/dL to 4.4-4.7 g/dL, while 24-hour urinary protein showed only a modest decline (from about 21 g to 18 g/day).

Urine protein electrophoresis was not performed because the test is not routinely available at our center, and in view of normal serum free light chains and κ/λ ratio and the absence of clinical or laboratory features of plasma cell dyscrasia, the pre-test probability of monoclonal proteinuria was considered low.

Despite this biochemical stabilization, she continued to experience intermittent yellow-white turbid urine with persistent 3+ dipstick proteinuria. Based on the overall clinical picture, the final working diagnosis was intermittent pseudochyluria with nephrotic-range proteinuria and preserved renal function in the setting of chronic bronchiectasis, managed with ongoing conservative therapy and regular clinical-biochemical follow-up.

## Discussion

This case highlights that milky urine with massive proteinuria does not automatically equate to primary glomerular disease or classic chyluria [1-3]. Negative chyle tests in repeated fasting samples, absence of filarial exposure or obstructive lymphatic pathology, and preserved serum albumin despite very high protein losses argue against overt chyluria [1,2]. Elevated urinary triglycerides in a fasting specimen confirm lipid-rich urine but are not pathognomonic for chyle and may also reflect lipiduria from nephrotic-range proteinuria [3]. The combination of intermittent white urine, raised urinary triglycerides, and negative chyle tests favours a mixed mechanism of lipid-rich nephrotic urine with possible low-grade lymphatic leakage that remains below the sensitivity of routine chyle assays [1-3].

In our patient, several features ultimately favoured pseudochyluria over frank chyluria despite the markedly elevated urinary triglycerides. All qualitative “chyle” assays, including early-morning fasting samples, repeatedly reported chyle as absent, and lymphocyturia was not documented. There was no demonstrable filarial infection, lymphatic obstruction, or retroperitoneal mass on imaging that could explain a lymphatico-urinary fistula. Clinically, the picture was dominated by massive glomerular proteinuria with subsequent normalization of serum albumin and stable renal function, which is compatible with lipid-rich nephrotic urine rather than sustained, high-volume chyle loss. The patient also noted that urine turbidity increased after oral protein supplements, suggesting that the “milky” appearance was at least partly driven by protein- and lipid-laden nephrotic urine. For these reasons, we used the pragmatic label of intermittent pseudochyluria on a background of nephrotic-range proteinuria, while acknowledging that a low-grade lymphatic leak cannot be completely excluded [1-3].

Chronic bronchiectasis prompted concern for AA amyloidosis, which can present with heavy proteinuria and progressive kidney dysfunction [4]. The underlying cause of her non-cystic fibrosis bronchiectasis could not be definitively established. The HRCT pattern of traction bronchiectasis with fibrotic bands and calcified mediastinal lymph nodes is most compatible with old post-infective disease (tuberculous or severe bacterial), and HIV, diabetes, and overt secondary immunodeficiency were excluded clinically and biochemically. However, a full etiologic workup for primary immunodeficiency, cystic fibrosis, primary ciliary dyskinesia, or allergic bronchopulmonary aspergillosis was not feasible in this resource-limited setting, and we acknowledge this uncertainty as a limitation. The combination of stable creatinine, normalized serum albumin, near-normal inflammatory markers, only minimally raised serum amyloid A, a non-diagnostic renal biopsy, and a negative fat-pad Congo red stain makes extensive, clinically overt systemic AA amyloidosis unlikely at present, while recognising that very early or focal AA disease cannot be fully excluded [4,5]. A normal serum amyloid A level reflects the absence of a strong ongoing inflammatory drive at the time of testing, but does not on its own rule out pre-existing AA deposits [4,5]. Likewise, negative serum free light chains with a normal κ/λ ratio reduce the likelihood of AL amyloidosis [6]. The absence of anti-PLA2R antibodies argues against classic primary membranous nephropathy, although other podocytopathies (including PLA2R-negative membranous nephropathy, minimal-change disease, or FSGS) cannot be conclusively distinguished without adequate renal tissue [5].

In resource-limited settings, where repeat biopsies, laser microdissection, and mass spectrometry are not readily available, a pragmatic approach combining urine chemistry (including urinary triglycerides), imaging, targeted serology (anti-PLA2R, serum free light chains), and low-risk tissue (fat-pad) biopsy can help narrow the differential. A key limitation of this report is the absence of a definitive histologic diagnosis, as further biopsies (repeat kidney or bladder) were not performed because of patient preference, perceived risk-benefit, and financial constraints. In addition, we could not obtain urine protein electrophoresis, which would have better characterised the protein profile, but was not feasible because of cost and limited local availability; however, the normal serum free light chain assay and clinical context made significant monoclonal proteinuria unlikely [6]. Our patient, therefore, exemplifies a real-world diagnostic dilemma at the interface of chyluria, pseudochyluria, and glomerular proteinuria, managed conservatively while avoiding unnecessary immunosuppression.

## Conclusions

This case highlights how intermittent “white urine” with nephrotic-range proteinuria and preserved renal function can pose a major diagnostic dilemma. Systematic evaluation excluded filarial chyluria, urinary tract obstruction, AA amyloidosis, AL amyloidosis, and primary membranous nephropathy, despite the patient’s background of chronic bronchiectasis and markedly elevated urinary triglycerides. We finally favoured a mixed mechanism of intermittent lipid-rich (pseudochylous) urine with an as-yet unclassified glomerular proteinuric disorder, managed conservatively with maximal antiproteinuric therapy and treatment of underlying bronchiectasis. The case underlines the need to approach “milky urine” analytically, to avoid prematurely labelling it as filarial chyluria or amyloidosis, and to integrate urine chemistry, imaging, limited histology, and serologic markers in resource-limited nephrology practice.
